# The Impact of Ecological Civilization Construction on Environment and Public Health—Evidence from the Implementation of Ecological Civilization Demonstration Area in China

**DOI:** 10.3390/ijerph19095361

**Published:** 2022-04-28

**Authors:** Zhifeng Zhang, Haodong Xu, Shuangshuang Shan, Yuqi Lu, Hongyan Duan

**Affiliations:** 1 School of Economics, Qingdao University, Qingdao 266071, China; sasha_china@hotmail.com; 2 School of Foreign Language Education, Qingdao University, Qingdao 266071, China; 3 School of Marxism, East China University of Political Science and Law, Shanghai 201620, China; anya.lu@outlook.com; 4 Department of Economics, The University of Sheffield, Sheffield S10 2TN, UK; hongyanduan7@gmail.com

**Keywords:** ecological civilization construction, ecological civilization demonstration area, environmental quality, public health, difference-in-difference model

## Abstract

Faced with an increasingly tight resource supply, serious environmental pollution and degrading ecosystems, human beings are eager to reduce environmental pollution and promote public health. In this context, this paper takes the ecological civilization demonstration area (ECDA) established in China as a quasi-natural experiment to test whether ecological civilization construction (ECC) is an effective solution for the reduction of environmental pollution and improvement of public health. Based on the panel data of 31 provinces in China from 2009 to 2020, the study analyzes the impact of ECC on environmental quality and public health by employing a difference-in-difference model. The results show that ECDA has restrained environmental pollution and reduced the morbidity and mortality, which indicates that ECC effectively promotes environmental quality and public health. The effect of ECC is more pronounced in economically developed regions. In addition, ECC improves environmental quality through scale effects, structural effects, technology effects, and ecological conservation effects, while the positive effects of ECC on public health are driven by scale effects and ecological conservation effects only. Therefore, policymakers should support low-carbon production, promote the upgrade of industrial structures, and encourage enterprises to develop green technologies. Ecological protection projects such as afforestation and greening are necessary. Governments should initiate ecological civilization construction in economically developed regions and then gradually promote the policies in relatively poor areas.

## 1. Introduction

Climate change and environmental pollution are global issues [1]. According to the World Health Organization (WHO), approximately 7 million people worldwide die from environmental pollution every year [2]. With the increase in human production activities, carbon dioxide emissions in various countries have been soaring, which poses a serious threat to environmental quality and human health [3,4,5]. China is the largest carbon emitter in the world [6], facing increasingly severe environmental pollution [7,8]. Environmental pollution, ecological environment damage, and other problems seriously threaten the health of Chinese residents [3]. Research shows that, in 2010, the number of premature deaths caused by environmental pollution in China reached approximately 1.2 million, accounting for approximately 40% of the total number of deaths in the world caused by environmental pollution [4].

Faced with increasingly serious environmental pollution and a decline in health levels [5,8,9], Chinese governments decided to carry out the ecological civilization construction (ECC), and upgraded the ECC to the “Millennium Plan”. In December 2013, six departments, including the National Development and Reform Commission, the Ministry of Finance, the Ministry of Land and Resources, the Ministry of Water Resources, the Ministry of Agriculture, and the State Forestry Administration, jointly issued the *Notice on Implementing the National Ecological Civilization Demonstration Area Construction Project (hereinafter referred to as the Notice)* (The *Notice on Implementing the National Ecological Civilization Demonstration Area Construction Project* originates from http://www.gov.cn/zwgk/2013-12/13/content_2547260.htm accessed on 7 March 2022). In June 2014, Jiangxi, Fujian, Guizhou, Yunnan, and Qinghai were selected as the first batch of provincial-level ecological civilization construction units (The documents and materials for the construction of ECDA come from the central government portal website (http://www.gov.cn/xinwen/2014-06/05/content_2694273.htm accessed on 7 March 2022). In terms of construction contents, the ecological civilization demonstration area (ECDA) advocates for the coordinated development of economy, politics, culture, society, and ecology at the macro level. Five aspects of the construction path, including ecological economy, ecological society, ecological environment, ecological culture, and ecological system, are determined at the micro level [10]. Table 1 shows the specific construction contents of ECDA.

**Figure 1:**
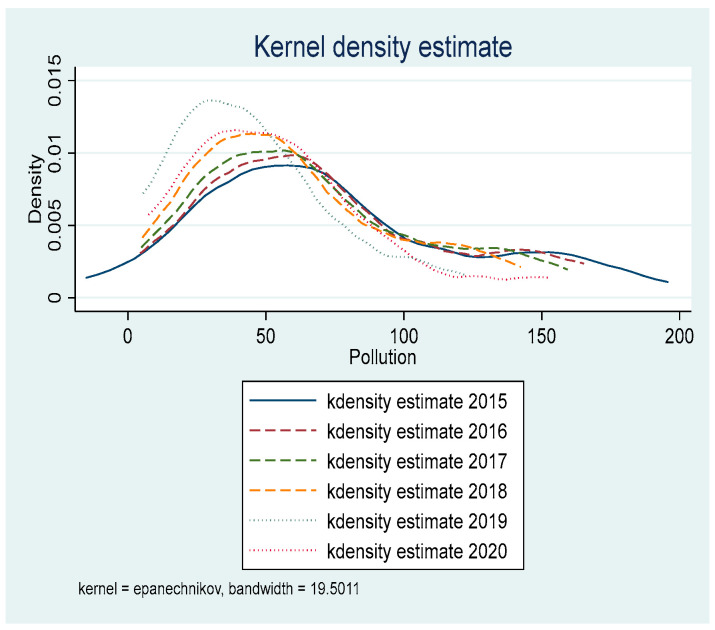
Dynamic evolution trend of environmental pollution.

**Figure 2:**
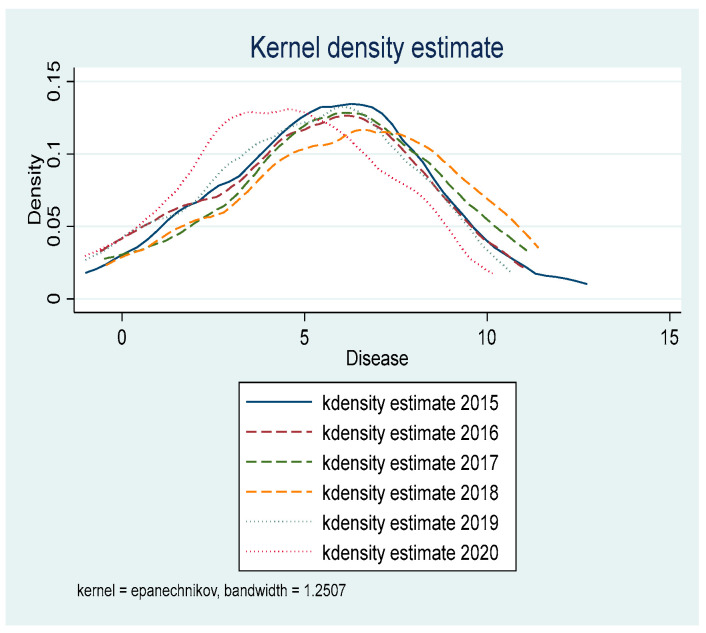
Dynamic evolution trend of the incidence of major diseases.

Figure 1 and Figure 2 present the kernel density curve of environmental pollution and incidence of major diseases among residents in ECDA-implemented provinces from 2015 to 2020. Kernel density estimation is a non-parametric estimation method of probability density function proposed by Rosenblatt [11]. Generally speaking, the dynamic evolution of an indicator over the years can be visualized in the form of a kernel density curve. Zhang et al. [12] sampled at the same time interval, and the sampling time interval was one year. Since the ECDA-implemented period of this paper is 2015–2020, the total six years of 2015, 2016, 2017, 2018, 2019, 2020 are sampled to plot the kernel density curve. Figure 1 and Figure 2 show that the peak of the kernel density curve keeps moving to the left and the density of the peak is increasing. Meanwhile, the degree of dispersion of the kernel density curves is becoming smaller. The above characteristics indicate that the pollutant emissions and the incidence of major diseases transform from low-level discreteness to high-level aggregation after the implementation of ECDA. Pollutant emissions and morbidity rate decrease and their values are more centralized. In addition, it can be found that the variation range of the kernel density curve in Figure 1 is larger than that in Figure 2, suggesting that the effect of ECDA on pollutant emissions is more obvious than the morbidity rate. This will be verified and explained in the mechanism analysis.

Using the ECDA implemented in China as a quasi-natural experiment, this paper analyzes the impact of ECDA on environmental quality and public health to test whether the ECC is an effective solution. The study aims to provide countries around the world with implications for policymakers to effectively mitigate environmental pollution and promote human health.

## 2. Literature Review and Theoretical Mechanism Analysis

### 2.1. Literature Review

Environment and public health issues have attracted extensive attention from academia for a long time [3,12,13]. This section analyzes the impact of ECC on environmental quality and public health. The literature related to this study is mainly divided into the following two aspects: firstly, the impact of ecological civilization construction on environmental quality; secondly, the impact of ecological civilization construction on residents’ health.

In essence, ecological civilization construction is composed of specific ecological environment policies. Due to the lack of literature on the impact of ecological civilization construction on environmental quality, this section mainly analyzes the studies of the impact of various environmental policies on environmental quality. On the one hand, some existing literature focuses on the impact of a certain environmental policy on a certain type of pollution. Macho-Stadler [14] compared the effects of pollution tax, pollutant discharge standards, and pollutant emission permits on the efficiency of environmental governance. The study showed that pollution tax was the most efficient system of environmental governance among the three methods. Li and Shen [15] empirically analyzed the impact of emission reduction subsidies and environmental protection loan systems on pollutant reduction, which indicated that the emission reduction effects of these two policies are not obvious. Qi et al. [16] found that the environmental petition system affected the pollution discharge of enterprises. Zeng et al. [17] classified different environmental policy tools, and empirically studied the impact of various environmental policy tools on different types of environmental pollution. Lin et al. [18] empirically studied the impact of environmental taxes on heavily polluting industries, and found that environmental taxes would reduce the pollutant emissions of highly polluting industries. Shen and Jin [19] analyzed the impact of the “River Chief System” on the environment and found that the system achieved preliminary effects of water pollution control without significantly reducing deep water pollutants. Wang et al. [2] empirically studied the impact of the Central Environmental Protection Supervision Policy (CEPSP) on air quality, and found that the implementation of the CEPSP has significantly improved air quality. Using the systematic GMM to examine the impact of environmental policies on the overall environmental quality in China, Zheng [20] found that environmental policies have a significantly positive impact on the improvement of environmental quality. On the other hand, some studies focus on the effects of environmental policies in accordance with different types of regulation. Market-based regulation and government subsidies can be effective in promoting environmental improvement and economic efficiency, while command-based regulation may lead to policy failure and thus have no significant effect on environmental improvement [21]. Mexico’s odd-even number restriction policy has worsened air quality and failed to achieve environmental improvements [22]. Chen et al. [23] found that when dealing with water pollution in the Yangtze River basin, the strict administrative control in the downstream area has led to a “pollution shelter effect”. Economic activities shifting to the upstream has actually aggravated the pollution. Tanaka [24] concluded that market-based environmental regulation improved environmental quality and reduced infant mortality by 20% in the experimental area. Based on the triple-differences method, Cai et al. [25] found that the government subsides restricted the inflow of foreign capital by taking the “double-control zones” policy as a quasi-natural experiment, while Chen et al. [26] concluded that the target-based evaluation system effectively reduces environmental pollution and promotes ecological protection. Tang et al. [27] conducted a study using sulfur dioxide $\left({\mathrm{SO}}_{2}\right)$ emissions and GDP growth rates and found that the implementation of environmental policies significantly controlled pollution emissions. However, there was a regional imbalance in this emission reduction, which led to the suppression of economic growth.

Little literature has directly studied the impact of ecological civilization construction on public health. The relevant literature mainly focuses on the impact of environmental quality on the health of residents. The health production function pioneered by Grossman [28] was an early theoretical model to study the impact of the environment on public health. Later scholars incorporated environmental factors into the health production function and further analyzed the impact of environmental factors on the health depreciation rate [29]. The studies on the relationship between environment and health are mainly classified into the following two aspects. On the one hand, some studies mainly analyzed the impact of environmental pollution on the health of residents from a socioeconomic or epidemiological perspective, which indicated that respiratory diseases, cardiovascular and cerebrovascular diseases, and heart disease are caused by severe environmental pollution [30,31]. Chay and Greenstone [32] analyzed the impact of the reduction of total suspended particulates on the health of American infants in the United States. They found that a 1% reduction in total suspended particulates (TSPs) resulted in a 0.35% decline in the infant mortality rate at the county level, implying that 2500 fewer infants died from 1980 to 1982 than would have in the absence of the TSP reductions. Maisonet et al. [33] used mortality as a surrogate indicator of the public health level to study the relationship between the environment and mortality, which indicated that environmental pollution had a significant impact on public health. The emission of sulfur dioxide $\left({\mathrm{SO}}_{2}\right)$ and ${\mathrm{PM}}_{10}$ (${\mathrm{PM}}_{10}$ refers to particulate matter that is less than or equal to 10 microns in diameter, also known as inhalable particulate matter) has increased the probability of residents suffering from respiratory diseases and lung cancer [34,35]. Xie et al. [36] argued that ${\mathrm{PM}}_{2.5}$ (${\mathrm{PM}}_{2.5}$ refers to particulate matter in the atmosphere that is less than or equal to 2.5 microns in diameter, also known as lung-accessible particulate matter) caused by industrial production significantly increases public additional health expenditures. Zhang et al. [37] analyzed the impact of environmental regulation on public health, regarding environmental pollution as the mediating variable. They concluded that environmental regulation promotes public health by improving environmental quality. Sun and Lu [38] found that air pollution would exacerbate health inequalities among residents based on the data from the China Comprehensive Social Survey. On the other hand, some scholars believe that the immature straw treatment caused air pollution. The traditional open-burning methods lead to a decline in air quality, and bring harmful gas to the ecological environment, thus deteriorating the health of residents to some extent [39,40]. Li and Jia [41] used the balanced panel datasets of the China Family Panel Studies (CFPS) to empirically test the negative impact of air pollution on public health and their differences among different groups. Ren et al. [42] and Lu and Qi [43] also drew a similar conclusion that air pollutants, such as sulfur dioxide $\left({\mathrm{SO}}_{2}\right)$, carbon dioxide$\;({\mathrm{CO}}_{2})$, ${\mathrm{PM}}_{2.5}$, and ${\mathrm{PM}}_{10},$ have significantly negative impacts on residents’ health.

### 2.2. Theoretical Mechanism Analysis

From a theoretical perspective, this section analyzes the policy mechanism of the establishment of ECDA affecting environmental quality and public health, which provides the theoretical basis for the mechanism analysis. Grossman and Krueger [44] concluded that the impact mechanism of an environmental policy on environmental pollution and public health can be separated into three factors: scale effect, structure effect, and technology effect. Yuan et al. [1], Zhang [45], and Li et al. [46] discussed the mechanism of the above three effects, respectively. As ECC advances in China, ecological conservation, human life, and public health are increasingly in demand. The ecological protection effect has become an important factor in environmental protection and health improvement, which are added into the study [47]. Referring to the above studies, this section expounds the impact mechanism of ECDA on environmental quality and public health based on the scale effect, structure effect, technology effect, and ecological protection effect. 

#### 2.2.1. Scale Effect

The scale effect is mainly reflected in the transformation of the extensive economic development model, which includes high energy consumption and high pollution, and the control of carbon dioxide emissions. This requires green transformation by promoting ecological economy construction everywhere, reducing pollutant emissions, saving energy, and recycling. The economic development of Jiangxi, Fujian, Guizhou, Yunnan, and Qinghai still lags behind that of other developed provinces, and they continue to inherit high-energy-consuming industries from other developed coastal areas [48]. It is shown that the development of a circular economy is an important method to reduce carbon emissions in heavily polluting industries [49]. The provincial-level units of ECDA have been accelerating the recycling transformation of industrial zones and the construction of the whole resource recycling system. The recycling of fossil energy is carried out vigorously to ensure the simultaneous reduction of fossil energy consumption such as coal, oil, and carbon emissions. Additionally, carbon dioxide emissions are controlled to improve the environmental quality and public health. It can be seen from the above analysis that, firstly, pollution reduction, energy saving, and recycling in ecological economic construction improve environmental quality and public health through the scale effect of carbon emissions. Secondly, carbon dioxide emission is an important indicator of the scale effect.

#### 2.2.2. Structure Effect

Inappropriate industrial structure is one of the most important factors causing the deterioration of the environment [6]. The share of the manufacturing industry has a positive effect on carbon emissions, while the services industry decreases gradually with the growth of its share. If the share of manufacturing disproportionately rises, the emissions of thermal power, coal consumption, and factory waste gas will increase suddenly, causing environmental deterioration [1]. Actively changing the current situation wherein economic growth is highly dependent on energy consumption and the secondary industry is an important strategy to reduce environmental pollution [50]. The relevant departments are required to accelerate the upgrading of the industrial structure, eliminate backward industries, and develop new industries in the construction of an ecological economy. Furthermore, ECDA raises the entry thresholds and constraints for heavily polluting and high-emission industries. More attention is paid to promoting strategic emerging industries towards green and high-quality models. ECDA requires the implemented provinces to shift the economic model from secondary industry to high-quality manufacture and tertiary industry, and transform the industrial structure from energy-intensive to knowledge-intensive. Therefore, the consumption of fossil energy and pollution emissions can be reduced to achieve the improvement of environmental quality and public health [1]. It can be seen from the above that, firstly, the upgradation of the industrial structure in ecological economic construction improves environmental quality and public health through the structure effect. Secondly, industrial structure is an important indicator of the structure effect.

#### 2.2.3. Technology Effect

Technological progress is the core driving force for reducing environmental pollution [51]. The technology effect refers to reducing the consumption of fossil energy and restraining environmental pollution by changing the production process and developing new energy technologies. The Notice requires that local authorities improve the process of clean production examination, new energy policy guarantee, and environmental protection approval in the construction of an ecological system. ECDA strengthens technological progress, and attaches importance to the innovation and upgradation of “clean” technology. For example, enterprises are encouraged to develop high-efficiency and energy-saving motors, and adopt new energy technologies such as electricity, wind, hydrogen, and natural gas energy to reduce carbon emissions. This leads to a dependence on “clean” technology, and the formation of a virtuous circle of pollutant emission reduction. In addition, the Notice requires that ECDA cultivates the awareness of environmental protection and cultural construction of enterprises in the construction of an ecological culture. By ecological culture construction, the innovation of green technology within enterprises can be promoted, and key technologies for reducing fossil energy pollution are developed rapidly [46]. Efforts should be taken to actively promote the low-carbonization of industrial industries, and constantly develop high-tech technology to promote the transformation from high-carbonization to low-carbonization economy. Therefore, pollutant emissions can be reduced and public health will be enhanced. It can be seen from the above analysis that, firstly, technological progress is an important manifestation of the technology effect. Secondly, both ecological system construction and ecological culture construction improve environmental quality and public health through the technology effect.

#### 2.2.4. Ecological Protection Effect

The ecological protection effect refers to the enhancement of the natural environment’s ability to absorb carbon pollutants by promoting the construction of nature reserves, forests, grasslands, and marine resources. The Notice clearly requires that the provinces in ECDA improve resource utilization and environmental protection in the ecological environment construction, such as afforestation and greening, ecological restoration, and environmental protection supervision. On the one hand, the afforestation and greening projects need to be implemented in depth, and the project of returning farmland to forest and grassland also continues to be promoted. Forest ecosystems play an important role in pollution reduction. The method of using forest carbon sequestration to offset greenhouse gas emissions has been adopted by most countries in the world [52]. On the other hand, the Notice emphasizes that local governments carry out relevant measures to promote people’s livelihood, education, and medical guarantee in the construction of an ecological society. ECDA improves people’s livelihoods by an ecological compensation mechanism and a series of ecological protection measures. Two conclusions can be drawn from the above: first, forest coverage is an important indicator of the ecological protection effect. Second, both ecological environment construction and ecological society construction promote environmental quality and public health through the ecological protection effect.

**Figure 3:**
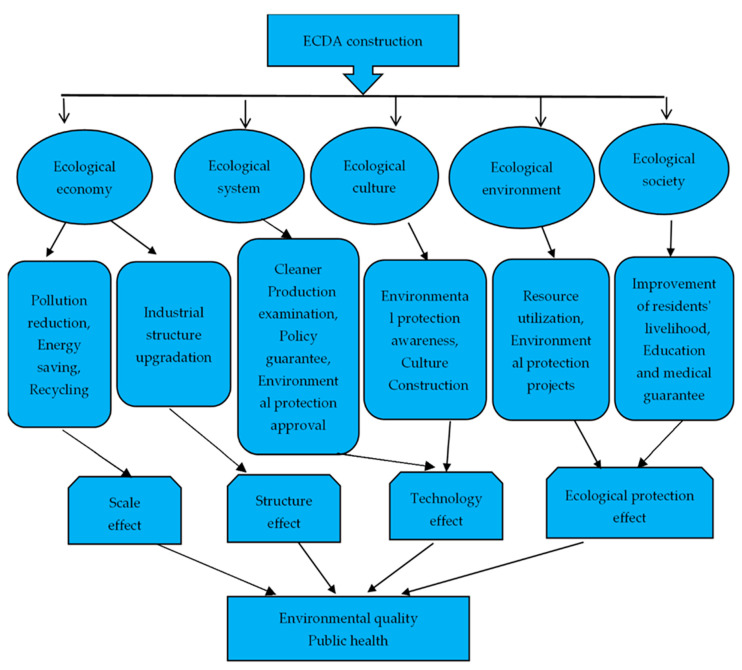
Theoretical framework.

Based on the above theoretical mechanism analysis, along with the specific construction contents of ECDA in Table 1, we construct the theoretical framework as shown in Figure 3, which provides the theoretical basis for the mediating effect analysis.

Compared with the existing studies, the innovations of this paper lie in the following three aspects. First, this study creatively combines environmental pollution and public health to examine the effectiveness of ECC. It overcomes the fact that previous literature analyzes the impact of environmental policies merely on environmental pollution or on public health. The second is the innovation of the research perspective. This paper provides global solutions for the protection of the ecological environment and the health improvement of the human population. The third is the innovation of the model design and methods. This paper employs the DID model, the triple-differences model, and mediation effect analysis to evaluate the policy effects [6,13]. The DID model is constructed for benchmark regression [53]. In order to verify the accuracy of the regression results, this paper conducts a series of robustness tests such as the parallel trend test, placebo test, and substitution of explained variables [54,55]. Triple-differences interaction terms are constructed to explore the heterogeneity characteristics of the effects of ECDA [55,56]. Furthermore, this study systematically sorts out the scale effect, structure effect, technology effect, and ecological protection effect. The stepwise regression test for coefficients, Sobel test, and bootstrap test [57,58] are used to test the internal mechanism of ECC on environmental quality and public health.

## 3. Data and Methods

### 3.1. Variables and Data

This paper selects the panel data of 31 provincial-level units (province for short) in China from 2009 to 2020, including 22 provinces, 5 autonomous regions, and 4 municipalities. Hong Kong Special Administrative Region, Macau Special Administrative Region, and Taiwan Province are not included in the research sample. The statistical software Stata (College Station, TX, USA) is used for empirical analysis. The data are obtained from the National Bureau of Statistics of China (http://www.stats.gov.cn/), China Environmental Yearbook (environmentcnki.net), Chinese Research Data Services Platform (https://www.cnrds.com/), CEADs database (www.ceads.net.cn), and CSMAR database (http://cndata1.csmar.com), all accessed on 7 March 2022. According to the relevant literature, environmental pollution is generally measured by air pollutant emissions [59,60].The incidence of major diseases issued by health institutions is generally used to represent public health [42,61]. Therefore, this study takes the emission of air pollutants (Pollution) and the incidence of major diseases (Disease) as the explained variables. Control variables include population density (Rpeo), total fixed asset investment (Inve), total retail consumption in the whole society (Scon), fiscal expenditure on environmental protection (Gov), economic development level (Eco), regional average education level (Edu), and urbanization rate (Urban) [6,12,13]. Variable definitions are shown in Table S1. In order to alleviate the influence of extreme values on empirical results, we winsorize all variables that are less than 1% of the quantile [7]. Table S2 shows descriptive statistics of the main variables in the study. Tables S1 and S2 are presented in the Supplementary Document (We would like to thank an anonymous reviewer for presenting us with the recommendation “*In general, well described. However, it could be short, and some items could transfer to supplementary documents*.” According to this suggestion, we have revised Section 3 and present Tables S1 and S2 in the Supplementary Document).

### 3.2. Research Methods and Model Design

This study attempts to analyze the impact of the ECDA on environmental pollution and public health, taking the ECDA in 2015 as a quasi-natural experiment. Referring to the studies of Zhang et al. [12], Li et al. [53], and Gao et al. [54], this paper adopts the difference-in-difference model for empirical regression, which is set as follows:

(1) $${\;Y}_{\mathrm{it}}=\alpha +\beta_{1}D_{\mathrm{it}}+\beta_{2}{\mathrm{control}}_{\mathrm{it}}+\lambda_{t}+\mu_{i}+\varepsilon_{\mathrm{it}}$$

where $Y_{\mathrm{it}}$ is the explained variable, including Pollution or Disease; Pollution and Disease are, respectively, used as the explained variables in the empirical analysis.$\;D_{\mathrm{it}}$ is the policy dummy variable of the DID model, which is the interaction term of ${\mathrm{treat}}_{i}$ and ${\mathrm{post}}_{t}$. ${\mathrm{Treat}}_{i}$ is the dummy variable regarding whether ECDA is established. Fujian, Jiangxi, Guizhou, Yunnan, and Qinghai are selected as the experimental group; hence, the value of ${\mathrm{treat}}_{i}$ is 1; otherwise, the value of ${\mathrm{treat}}_{i}$ is 0. ${\mathrm{Post}}_{t}$ is a dummy variable for time, representing the time when ECDA takes effect. ECDA was formally implemented in June 2014 and the duration in 2014 was less than one year, so we held that ECDA came into effect in 2015. In 2015 and after, the value of ${\mathrm{post}}_{t}\;$is 1; otherwise, the value of ${\mathrm{post}}_{t}$ is 0. $\beta_{1}$ is the core indicator to measure the effect of the implementation of ECDA. ${\mathrm{Control}}_{\mathrm{it}}$ represents the control variables, $\lambda_{t}$ is the year-fixed effect, $\mu_{i}$ is the province-fixed effect, and $\varepsilon_{\mathrm{it}}$ is the random error term.

## 4. Results

### 4.1. Benchmark Regression Analysis

Table 2 presents the results of the benchmark regression analysis. Columns a and c include no control variables. According to the stepwise regression method, control variables are added in columns b and d. Columns a and b examine the causal relationship between ECDA and environmental pollution. In columns a and b, the coefficient of $D_{\mathrm{it}}$ is −9.786 and −7.658, respectively, both of which are negative at the 1% significance level. The results of columns a and b suggest that ECDA has reduced pollutant emissions and improved environmental quality. Columns c and d test the causal effect of ECDA on public health. In column c, the coefficient of $D_{\mathrm{it}}$ is −0.088, which is not significant. Column d adds control variables on the basis of column c. In column d, $\beta_{1}$ is −0.220, which is significantly negative at the 10% significance level. The results of columns c and d show that ECDA has reduced the incidence of major diseases and promoted residents’ health. It can be concluded that ECDA improves the environmental quality and the health of residents. ECC is therefore an effective solution not only for mitigating environmental protection, but also for human health improvement.

### 4.2. Robustness Test

This study uses three methods to verify the accuracy of the regression results. The first method is the parallel trend test. The second method is the placebo test. The third is the replacement of the explained variables. In the third method, the explained variables in model (1) are replaced by the number of environmental incidents and the mortality rate, respectively [12,54,55].

#### 4.2.1. Parallel Trend Test

**Figure 4:**
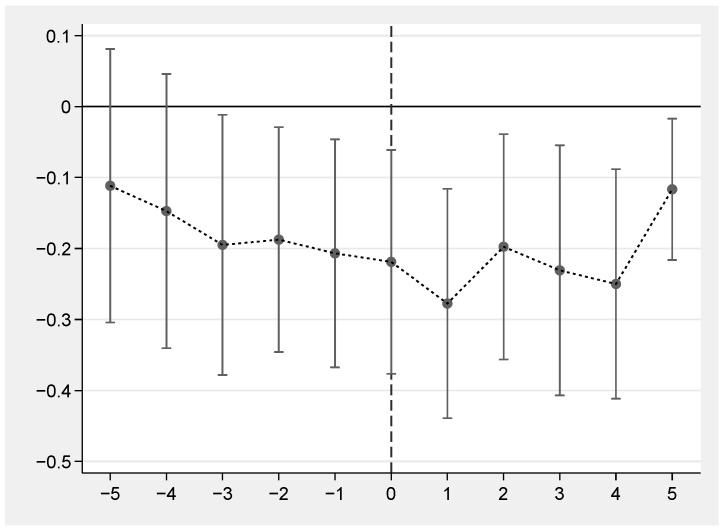
Parallel trend test.

One of the most important assumptions of the difference-in-difference model is that the parallel trend assumption is satisfied. The parallel trend assumption means that before the implementation of the policy, the experimental group and the control group should have the same variation trend. Referring to related studies [54,62,63], this section constructs the two-way fixed-effect model, as shown in model (2).

(2) $$Y_{\mathrm{it}}=\alpha +\beta_{i}\overset{5}{\underset{k=-5}{\sum }}D^{k}{}_{\mathrm{it}}+{\tau \mathrm{control}}_{\mathrm{it}}+\lambda_{t}+\mu_{i}+\varepsilon_{\mathrm{it}}$$

where the value of k is based on the year when ECDA comes into effect. Specifically, 2015 is the effective start year of the ECDA, so the value of k in 2015 is 0. For provinces during k years after ECDA is implemented, the value of $D^{k}{}_{\mathrm{it}}$ is 1; otherwise, the value of${\;D}^{k}{}_{\mathrm{it}}$ is 0. The value of k ranges from −5 to 5, and the meanings of other variables are consistent with model (1). Figure 4 shows that before the implementation of ECDA, there was no obvious trend difference in the incidence of major diseases between the experimental group and the control group. However, the incidence of major diseases began to decrease significantly after the implementation of ECDA, indicating that ECDA has a significant impact on public health. This result proves that model (1) conforms to the parallel trend assumption. Therefore, the difference-in-difference model is effective.

#### 4.2.2. Placebo Test

**Figure 5:**
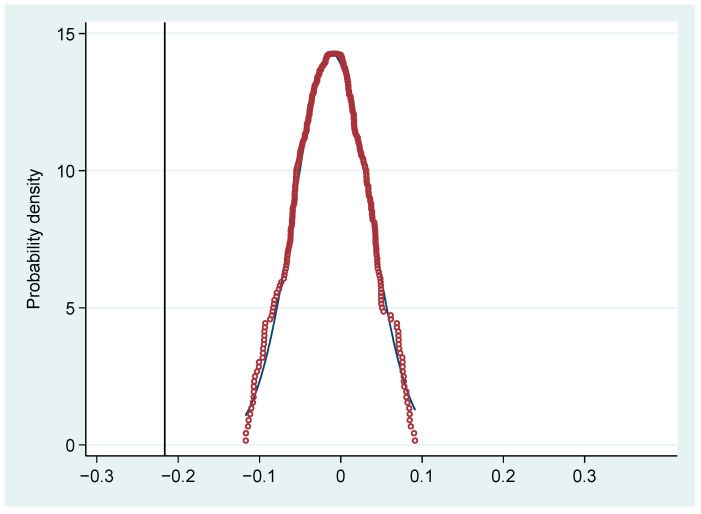
Placebo test.

The basic idea behind the placebo test is to estimate the benchmark model based on the dummy treatment group or the dummy policy time. If the coefficient of the “pseudo-policy dummy variable” is still significant in the fictitious situation, the original estimation results are likely to be biased [7]. This section conducts a placebo test referring to Lu et al. [64], in order to prove that the changes in environmental pollution and public health in ECDA-implemented provinces are caused by ECC rather than other unobservable factors. Specifically, we randomly select some provinces from the total samples as the treatment group to re-estimate model (1). This process is repeated 1000 times, so that 1000 coefficient estimation results can be obtained, from which the kernel density graph is drawn. As shown in Figure 5, the probability density graph is expected to follow a normal distribution, and the coefficient estimates obtained above are obviously different from the mean value of the kernel density distribution. This test proves that the causality effects of ECC on environmental pollution and residents’ health are not derived from other unobservable factors but indeed come from the implementation of ECDA itself.

#### 4.2.3. Replacement of the Explained Variables

In order to verify the effects of ECC on environmental pollution and public health, this section replaces the explained variables for the robustness test. The study replaces air pollutant emissions and the incidence of major diseases with the number of environmental incidents (Environ) and the mortality rate (Death), respectively [12,41,61]. The number of environmental incidents refers to the amount of incidents that occur suddenly, cause heavy casualties and severe property losses, or threaten and damage the economic and social stability of the country in one year [40,41]. It is an important indicator to measure environmental pollution. Table 3 shows the descriptive statistics of the surrogate variables.

The results of replacing the explained variables are shown in Table 4. In columns a and b, the coefficients of $D_{\mathrm{it}}$ are −13.100 and −5.099, respectively. The coefficients are not significant when no control variables are added; however, they are significant at the 5% significance level after the control variables are added. In columns c and d, the coefficients of $D_{\mathrm{it}}$ are −0.191 and −0.191, both significant at the 10% significance level. The results suggest that after the implementation of ECDA, the number of environmental events and the mortality rate have decreased, regardless of whether control variables are added or not. ECC has reduced environmental pollution, improved environmental quality, and promoted public health. This indicates that the results of the benchmark regression are robust.

### 4.3. Heterogeneity Analysis

Considering the great differences in economic development, industrial structure, resource utilization, and environmental pollution among different regions in China, the policy effects of ECDA may have regional heterogeneity [65]. This paper conducts the heterogeneity analysis of ECDA in different regions, in order to test whether there is regional heterogeneity in the impact of ECC on environmental pollution and public health. This study classifies the samples into eastern, central, and western regions in China, referring to the division in the study of Yuan et al. [66]. Considering that the number of provinces in the western region is too small to compare with the central and eastern regions, we merge the central and western regions. Furthermore, we introduce the grouping dummy variable ${\mathrm{Group}}_{\mathrm{it}}$ to represent regional differences. For provinces in the eastern region, the value of ${\mathrm{Group}}_{\mathrm{it}}$ is taken as 1; otherwise, ${\mathrm{Group}}_{\mathrm{it}}=$ 0. Therefore, the triple-differences model is constructed by introducing the interaction term between $D_{\mathrm{it}}$ and ${\mathrm{Group}}_{\mathrm{it}}$ into model (1) [55,56], as shown in model (3). The meanings of other variables are consistent with model (1).

(3) $${\;Y}_{\mathrm{it}}=\alpha +\beta_{1}D_{\mathrm{it}}+\beta_{2}(D_{\mathrm{it}\;}\times {\;\mathrm{Group}}_{\mathrm{it}\;})+\beta_{3}{\mathrm{control}}_{\mathrm{it}}+\lambda_{t}+\mu_{i}+\varepsilon_{\mathrm{it}}$$

The result of the heterogeneity analysis is presented in Table 5. According to the stepwise regression method, control variables are added in the columns b and d on the basis of columns a and c, respectively. It can be seen that the coefficients of $D_{\mathrm{it}}\times {\mathrm{Group}}_{\mathrm{it}}$ are significantly positive, regardless of whether the control variable is added or not. The coefficients of column a and column b are 4.843 and 0.999, which are significant at the 5% level. The coefficient of column c is 0.503, which is significant at the 1% level. The coefficient of column d is 0.162, which is significant at the 10% level. The results show that compared with the central and western regions, the implementation of ECDA in the eastern region has a more significant effect on improving environmental quality and public health. This may be attributed to the relatively developed economy and high industrial output value in the eastern region. In addition to daily production and operation, there are enough funds to develop clean production and green technology innovation [1]. Cleaner production industry can be established in a short period of time, actively responding to the implementation of ECDA [34]. The eastern region always indicates economically developed regions, while the central and western regions represent relatively poor regions [12]. Therefore, it is concluded that the effects of ECDA on reducing environmental pollution and improving public health are more pronounced in economically developed regions.

### 4.4. Mechanism Analysis

This paper uses the mediation effect to analyze the mechanism of ECDA affecting environmental quality and public health. Based on the theoretical mechanism analysis, carbon dioxide emissions (Carbon), industry ratio (Stru), technological progress rate (Tech), and forest coverage rate (Forest) are selected as mediating variables. Among them, carbon dioxide emissions (Carbon) represents the scale effect, industry ratio (Stru) represents the structure effect, technological progress rate (Tech) represents the technology effect, and forest coverage rate (Forest) represents the ecological protection effect. Referring to the related literature [12,57], the mediation effect model is as shown below, including Formula (4).

(4) $$\{\begin{array}{l} Y_{it}=cD_{it}+\delta_{1}{\mathrm{control}}_{\mathrm{it}}+e_{1}\;\;\;\;\;\;\;\;\;\;\;\;\;\;\;\;\;\;\;\;\;\;\;\\ M_{it}=aD_{it}+\delta_{2}{\mathrm{control}}_{\mathrm{it}}+e_{2}\;\;\;\;\;\;\;\;\;\;\;\;\;\;\;\;\;\;\;\;\;\\ Y_{it}=c^{\prime }D_{it}+bM_{it}+\delta_{3}{\mathrm{control}}_{\mathrm{it}}+e_{3}\;\;\;\; \end{array}$$

where $Y_{it}$ is the explained variable, including environmental pollution (Pollution) and the incidence of major diseases (Disease). $M_{it}$ is the mediator, including carbon dioxide emissions (Carbon), industry ratio (Stru), technological progress rate (Tech), and forest coverage (Forest). $D_{it}$ represents the difference-in-difference interaction term. c represents the total effect of the implementation of ECDA on $Y_{it}$, while c′ represents the direct effect, and ab represents the indirect effect, namely the mediating effect. The total effect is the sum of the direct effect and the indirect effect. Table 6 shows the descriptive statistics of the mediating variables. 

In this study, the stepwise regression test of coefficients, Sobel test, and bootstrap test [57,58,67] are used to verify the scale effect, structure effect, technology effect, and ecological conservation effect. There are three steps in the stepwise regression test for coefficients [57]. The first step is to test the coefficient c in the model (4), the second step is to test the coefficient a in the model (5), and the third step is to test coefficients c′ and b in the model (6). If the coefficients a, b, and c are all significant, the mediation effect is valid. If one of the coefficients is not significant, the stepwise regression test for coefficients fails to examine the mediation effect [58]. Since some coefficients are not significant, the stepwise regression test for coefficients is invalid. Due to the limited space, this study will not show the results of the stepwise regression test for coefficients. Therefore, this study further uses the Sobel test to analyze the mediation effect, of which the test power is higher than the stepwise regression test for coefficients [68]. 

Table 7 shows the results of the Sobel test. First, we analyze the impact mechanism of ECDA on environmental pollution. It can be found in the study that the *p* values of the scale effect, structure effect, technology effect, and ecological protection effect are all less than 0.05. Therefore, the mediation effect of ECDA on environmental pollution is valid, indicating that ECC reduces environmental pollution through the scale effect, structure effect, technology effect, and ecological protection effect. The mediating effects of the above four paths account for 8.73%, 14.65%, 13.72%, and 23.58%, respectively. Second, we examine the mechanism of ECDA on public health. The study shows that the *p* values of the scale effect and ecological protection effect are less than 0.05, while the *p* values of the structure effect and technology effect are both greater than 0.05. The results suggest that the impact of ECDA on the improvement of public health is effective through the scale effect and ecological protection effect, while the technology effect and structure effect are not valid. The mediating effect of the scale effect and ecological protection effect accounts for 3.88% and 50.73%, respectively. To sum up, ECC improves environmental quality by means of the following four paths: the scale effect, structure effect, technology effect, and ecological protection effect. However, ECC promotes residents’ health by means of the scale effect and ecological protection effect, and the mediating effect of the ecological protection effect is dominant. Based on the mediation effect analysis, it can be clearly seen that there are four mechanism paths for ECDA to affect environmental quality, but only two mechanism paths in terms of public health. Therefore, the effect of ECC on environmental quality will be more obvious than the effect of improving the health of residents, which confirms the findings shown in Figure 1 and Figure 2.

In addition, this section adopts the bootstrap test to verify the results of the mediation effect analysis mentioned above [12]. We use random sampling of 1000 times to conduct the bootstrap test. The results of bootstrap random sampling are the estimated values of the coefficient products, and sorted from small to large, where the 2.5th percentile and the 97.5th percentile form a confidence interval at the 95% confidence level. The mediation effect is valid if the confidence interval does not contain 0; otherwise, the mediation effect does not hold [69,70]. The result of the bootstrap test is presented in Table 8. In terms of the impact mechanism of ECDA on environmental pollution, the confidence intervals of the scale effect, structure effect, technology effect, and ecological protection effect do not contain 0. For the impact mechanism of ECDA on public health, the confidence intervals of the scale effect and ecological protection effect do not contain 0, while the confidence intervals of the structure effect and technology effect both contain 0. To sum up, the results of the bootstrap test have verified the conclusions drawn from the Sobel test.

## 5. Discussion

This study makes a significant contribution to exploring the impact of ECC on environmental quality and public health. Taking the ECDA implemented in China as a study case, we find that ECC effectively promotes environmental quality and public health, which is consistent with the conclusions of Xie et al. [13]. However, the methods are quite different. Xie et al. [13] used the DID model to explore the impact of ECDA on air pollution, and used the synthetic control method to analyze the policy effect on public health. Instead, this paper adopts the DID model, triple-differences model, and mediation effect methods to analyze the effects of ECDA. In terms of the impact mechanism, this paper tests the scale effect, structure effect, technology effect, and ecological protection effect by using mediation effect methods. The mediating effect analysis is similar to Liu [6], both of which verify the scale effect, structure effect, technology effect, and ecological protection effect. In the heterogeneity analysis, this study tests the regional heterogeneity characteristics of ECDA, among the eastern, central, and western regions in China. It is also verified in the studies of Xie et al. [13]. Additionally, this paper aims to provide global solutions for policymakers to reduce carbon emissions, protect the ecological environment, and improve residents’ health, while the findings of Mi et al. [9] fail to provide solutions from a global perspective. The study of Mi et al. [9] evaluated the effectiveness of ECC based on the data of Jiangsu Province in China and provided guidance for the optimization of ecological civilization policies merely in Jiangsu Province. Compared with the studies of Liu [6], Mi et al. [9], and Xie et al. [13], this paper provides new evidence and implications for addressing global environment and public health issues.

## 6. Conclusions 

The results of the study show that ECDA has restrained environmental pollution, and reduced morbidity and mortality, which indicates that ECC effectively promotes environmental quality and public health. The effect of ECC is more pronounced in economically developed regions. Furthermore, ECC improves environmental quality by means of the scale effect, structure effect, technology effect, and ecological protection effect, while the positive effect of ECC on public health is only driven by the scale effect and ecological protection effect.

The conclusions of this study provide solutions for policymakers around the world to effectively reduce pollutant emissions, protect the ecological environment, and promote human health, especially applicable to some developing countries such as China. From the perspective of the scale effect, structure effect, technology effect, and ecological protection effect, governments around the world should formulate policies to support low-carbon production, promote the upgrade of the industrial structure, and develop a cleaner production industry. Meanwhile, authorities should encourage green technology innovation in enterprises and provide support for new energy technologies to realize green production. In addition, ecological protection projects such as afforestation and greening are necessary. The environmental protection sectors should strengthen ecological restoration and protection, so as to increase the absorption of air pollutants. From the perspective of regional heterogeneity, governments should initiate ecological civilization construction in economically developed regions, and then gradually promote the policies in relatively poor regions.

There are several limitations and future directions to fulfill in this study. First, we fail to examine the mediating effect of environmental pollution on public health. Zhang [37] used environmental pollution as an intermediary variable to test the causal relationship between environmental regulation policies and public health. It is worth taking into account the impact of environmental pollution on public health in future research. Second, we only analyze the regional heterogeneity, without considering other heterogeneity characteristics in the empirical research. Future research will focus on a series of heterogeneous environmental policies to test their effects on environmental quality and public health.

## Figures and Tables

**Table 1:** The construction contents of ECDA.

First-Level Indicators	Secondary-Level Indicators	Contents
Ecological economy	Industrial structure upgradation	Within the carrying capacity of the ecosystem, policymakers use the principles of ecological economics and the methods of systematic engineering to change production and consumption patterns, exploit all available resource potentials, develop economically and ecologically efficient industries, and eventually achieve a sustainable development model in which economic growth and environmental protection, material civilization and spiritual civilization, natural ecology, and human ecology are highly unified.
	Pollution reduction	
	Energy saving, Recycling	
Ecological society	Improvement of residents’ livelihood	Based on rational use of resources and prevention of environmental pollution, social ecology is achieved in education, medical care and improvement of people’s livelihood, and a living state in which human society and natural environment are integrated.
	Education and medical guarantee	
Ecological environment	Resource utilization	Ecological environment mainly focuses on resource utilization and environmental protection. Resource utilization includes the protection of non-renewable natural resources such as water, soil, and air and renewable natural resources. The society of mankind eventually achieves the effect of environmental ecology by rational resource utilization. The contents of environmental protection include forest coverage, water quality compliance, harmless treatment of domestic waste, urban green coverage compliance, sewage treatment, soil erosion control, etc.
	Environmental protection projects	
Ecological culture	Environmental protection awareness	The contents of ecological culture are not only to shape the cultural awareness of residents in a region in the long-term life and production process, but also to clarify the concept of green innovation, appropriate utilization, and protection of resources and environment.
	Culture construction	
Ecological system	Policy guarantee Cleaner production examination Environmental protection approval	Ecological system is to build an institutional system in line with the ecological civilization construction, including the performance evaluation system, reward mechanism and punishment measure for ecological civilization governance, and compensation system for ecological civilization construction. The specific manifestations include tax reduction, preferential policies, promotion and other incentive measures for environmentally friendly and clean production enterprises, and punishment measures such as circular criticism, tax increase, forced delisting, and entry into the negative list for heavily polluting enterprises.

**Table 2:** Benchmark regression.

Variable	Pollution		Disease	
	(a)	(b)	(c)	(d)
D	−9.786 ***	−7.658 ***	−0.088	−0.220 *
	(2.886)	(2.495)	(0.264)	(0.245)
Rpeo		1.624		0.735 **
		(1.551)		(0.332)
Inve		3.724 ***		0.072
		(1.004)		(0.188)
Scon		4.356		0.188
		(2.689)		(0.143)
Gov		−7.286 ***		−0.627
		(1.519)		(0.445)
Eco		4.891		−0.321
		(4.747)		(0.557)
Edu		−6.701		−0.384
		(12.419)		(0.708)
Urban		0.091		0.084
		(0.965)		(0.073)
Cons	77.557 ***	61.948	5.465 ***	0.365
	(1.686)	(48.447)	(0.133)	(2.530)
Province-fixed effect	Control	Control	Control	Control
Year-fixed effect	Control	Control	Control	Control
Observations	217	190	372	288
R-squared	0.642	0.718	0.722	0.726

Notes: The parentheses indicate the clustered standard errors at the prefecture-level province level. ***, **, and * indicate significance at the 1%, 5%, and 10% levels, respectively.

**Table 3:** Descriptive statistics of surrogate variables.

Statistic	Variable	Unit	Observations	Mean	Standard Deviation	Min	Max
Environ	Number of environmental incidents	Number of times	270	13.6777	26.5369	1	250
Death	Mortality rate	%	310	2.41126	1.89064	0.1	8.92

**Table 4:** Robustness test.

Variable	Environ		Death	
	(a)	(b)	(c)	(d)
D	−13.100	−5.099 **	−0.191 *	−0.203 *
	(8.104)	(4.656)	(0.112)	(0.109)
Rpeo		3.762 **		0.140 ***
		(3.890)		(0.027)
Inve		1.700 *		0.339 ***
		(2.131)		(0.056)
Scon		12.560		0.043
		(8.536)		(0.181)
Gov		−0.025 *		−0.476 ***
		(0.056)		(0.075)
Eco		6.900 *		0.631 ***
		(5.545)		(0.106)
Edu		−20.643		−0.066
		(24.288)		(0.431)
Urban		5.264 ***		0.064
		(3.966)		(0.039)
Cons	16.533 ***	−154.234	1.883 ***	−2.125
	(4.274)	(141.685)	(0.082)	(1.968)
Province-fixed effect	Control	Control	Control	Control
Year-fixed effect	Control	Control	Control	Control
Observations	270	244	310	257
R-squared	0.664	0.671	0.566	0.595

Notes: The parentheses indicate the clustered standard errors at the prefecture-level province level. ***, **, and * indicate significance at the 1%, 5%, and 10% levels, respectively.

**Table 5:** Heterogeneity analysis.

Variable	Pollution		Disease	
	(a)	(b)	(c)	(d)
D	−10.755 ***	−7.432 **	−0.188	−0.256 *
	(2.962)	(3.197)	(0.275)	(0.284)
D × group	4.843 **	0.999 **	0.503 ***	0.162 *
	(1.832)	(4.446)	(0.201)	(0.261)
Rpeo		3.724 ***		0.188 *
		(1.006)		(0.144)
Inve		1.625		0.734 **
		(1.558)		(0.333)
Scon		7.286 ***		0.072
		(1.522)		(0.188)
Gov		−0.060 ***		−0.516 **
		(0.022)		(0.355)
Eco		4.355		−0.277
		(2.699)		(0.216)
Edu		−6.822		−0.398 **
		(12.798)		(0.714)
Urban		0.096		0.081
		(0.973)		(0.073)
Cons	77.557 ***	61.471	5.465 ***	3.414
	(1.685)	(49.857)	(0.133)	(2.542)
Province-fixed effect	Control	Control	Control	Control
Year-fixed effect	Control	Control	Control	Control
Observations	217	190	372	288
R-squared	0.643	0.718	0.524	0.626

Notes: The parentheses indicate the clustered standard errors at the prefecture-level province level. ***, **, and * indicate significant at the 1%, 5%, and 10% levels, respectively.

**Table 6:** Descriptive statistics of mediating variables.

Statistic	Variable	Unit	Observations	Mean	Standard Deviation	Min	Max
Carbon	Carbon dioxide emissions	Metric ton	372	341.17	273.348	32.12	1700.04
Stru	Industry ratio	-	341	4.8253	67.4812	12.33	56.186
Tech	Technological progress rate	%	364	2.6711	15.5041	0.0321	212.33
Forest	Forest coverage	%	372	33.565	18.1999	4.2	66.8

**Table 7:** Sobel test.

Mechanism	Pollution		Disease	
	*p*-Value	The Proportion of Mediation Effect	*p*-Value	The Proportion of Mediation Effect
Scale effect	0.0235	8.73%	0.0475	3.88%
Structure effect	0.0461	14.65%	0.2837	7.91%
Technology effect	0.0348	13.72%	0.5334	3.59%
Ecological protection effect	0.0306	23.58%	0.0013	50.73%
Control variables	Control	Control	Control	Control
Province-fixed effect	Control	Control	Control	Control
Year-fixed effect	Control	Control	Control	Control

**Table 8:** Bootstrap test.

Mechanism	Pollution	Disease
	Confidence Interval	Confidence Interval
Scale effect	[−39.4556, −18.9518]	[0.5010, 2.0477]
Structure effect	[−38.0272, −15.0994]	[−3.1621, 2.0730]
Technology effect	[−40.9196, −22.3594]	[−0.5310, 2.0642]
Ecological protection effect	[−33.5607, −10.6868]	[1.1286, 2.5698]
Control variables	Control	Control
Province-fixed effect	Control	Control
Year-fixed effect	Control	Control

## Data Availability

Not applicable.

## Supplementary Materials

The following supporting information can be downloaded at: https://www.mdpi.com/article/10.3390/ijerph19095361/s1, Table S1: Variable description; Table S2. Descriptive statistics.
