# Supplementary material for: The Impact of Ecological Civilization Construction on Environment and Public Health—Evidence from the Implementation of Ecological Civilization Demonstration Area in China
Source: Int J Environ Res Public Health. 2022 Apr 28;19(9):5361. doi: 10.3390/ijerph19095361 (PMC9100575; doi:10.3390/ijerph19095361)
Supplement: Supplementary file 1 [file ijerph-19-05361-s001.zip › supplementary documentú¿associate with manuscriptú⌐.pdf]

Supplementary document

We would like to thank an anonymous reviewer presenting us with the recommendation *"In general, well described. However, it could be short, and some items could transfer to supplementary documents."* According to this suggestion, we revise section 3 and present Table S1 and Table S2 in the supplementary document.

Table S1. Variable description.

Variable Type	Variables	Statistic	Variable Definition
Explained variables	Air pollutant emissions	Pollution	Emissions of harmful gases
	Incidence of major diseases	Disease	Number of people suffering from major diseases defined by national health agency standards/ total population
Explanatory variables	Interaction term of difference-in-difference	D (Treat × post)	Five provinces of Fujian, Jiangxi, Guizhou, Yunnan, and Qinghai are the experimental group, and the remaining 26 provinces are the control group. Treat is taken as 1 in the experimental group, otherwise treat = 0. Post is taken as 1 in 2015 and later, post = 0 before 2015
Control variables	Population density	Rpeo	The ratio of permanent resident population to area at the end of each year in each province
	Total fixed asset investment	Inve	Including investment by state-owned economic units, investment by urban and rural collective economic units, and individual investment by urban and rural residents
	Total retail consumption in the whole society	Scon	The amount of non-production and non-operating physical commodities sold by enterprises in each province to individuals and social groups through transactions, as well as the amount of income obtained by providing catering services

	Environmental fiscal expenditure	Gov	Local fiscal expenditures for environmental protection
	The level of economic development	Eco	GDP per capita
	Regional average education level	Edu	(6×P elementary school+9×P junior high school+12×P high school+16×P college or above)/ (P elementary school + P junior high school + P high school + P college or above), P represents the educated population of each degree
	Urbanization rate	Urban	The proportion of urban population in total population at the end of each year

Table S2. Descriptive statistics.

Statistic	Unit	Observations	Means	Standard Deviation	Min	Max
Pollution	Thousand tons	217	644.029	425.9463	40.6	1801.1
Disease	%	372	5.340511	2.796107	-1.01	11.47
D	-	372	0.078947	0.270012	0	1
Rpeo	Person/square kilometer	372	2828.538	1178.564	515	5821
Inve	Billion CNY	341	1234.145	1050.123	27.034	5520.272
Scon	Billion CNY	372	904.9052	820.8304	15.66	4295.18
Gov	Billion CNY	372	13.30051	9.689928	0.975	74.744
Eco	CNY	372	42258.07	27133	3.2931	140211.2
Edu	%	372	2.487075	0.890895	0.969	6.75
Urban	%	319	54.79279	13.78885	22.6	89.6
